# Deformable 96-well cell culture plate compatible with high-throughput screening platforms

**DOI:** 10.1371/journal.pone.0203448

**Published:** 2018-09-06

**Authors:** Tsubasa S. Matsui, Hugejile Wu, Shinji Deguchi

**Affiliations:** 1 Division of Bioengineering, Graduate School of Engineering Science, Osaka University, Toyonaka, Osaka, Japan; 2 Department of Nanopharmaceutical Sciences, Nagoya Institute of Technology, Nagoya, Aichi, Japan; Universitatsklinikum Freiburg, GERMANY

## Abstract

Adherent cells such as endothelial cells sense applied mechanical stretch to adapt to changes in their surrounding mechanical environment. Despite numerous studies, signaling pathways underlying the cellular mechanosensing and adaptation remain to be fully elucidated partly because of the lack of tools that allow for a comprehensive screening approach. Conventionally, multi-well cell culture plates of standard configurations are used for comprehensive analyses in cell biology study to identify key molecules in a high-throughput manner. Given that situation, here we design a 96-well cell culture plate made of elastic silicone and mechanically stretchable using a motorized device. Computational analysis suggested that highly uniform stretch can be applied to each of the wells other than the peripheral wells. Elastic image registration-based experimental evaluation on stretch distributions within individual wells revealed the presence of larger variations among wells compared to those in the computational analysis, but a stretch level of 10%–that has been employed in conventional studies on cellular response to stretch—was almost achieved with our setup. We exposed vascular smooth muscle cells to cyclic stretch using the device to demonstrate morphological repolarization of the cells, i.e. typical cellular response to cyclic stretch. Because the deformable multi-well plate validated here is compatible with other high-throughput screening-oriented technologies, we expect this novel system to be utilized for future comprehensive analyses of stretch-related signaling pathways.

## Introduction

Cells sense mechanical stretch to regulate their morphology and function [1]. Cellular response to stretch has been demonstrated to play a pivotal role in developmental process [2,3] as well as in health maintenance [4]. One example includes the behavior of endothelial cells that orients their morphological polarization into a direction perpendicular to the direction of stretch upon heartbeat-induced cyclic stretch [5–8]. This endothelial cellular response to orient away from stretch has been implicated in suppression of atherosclerosis to circumvent prolonged activation of pro-inflammatory signals [5,9,10].

Not limited to endothelial cells, stretch sensing capability is widely conserved among various mammalian cell types, and thus underlying mechanisms have been extensively studied by identifying signaling pathways responsible for the cellular response to stretch [7,11–15]. The use of high-throughput analytical tools is often key to allowing comprehensive identification of such molecules at the genome-wide level [16]. High-throughput systems typically adopt a multi-well plate format designed according to internationally standardized geometric configurations and thus compatible with available imaging systems such as automated fluorescence plate readers [17]. Besides, the universal design with the standard geometry enables direct coupling with siRNA/shRNA/drug libraries that are supplied as the same multi-well plate format. Although various new methodologies have been proposed to study cellular response to stretch [18–21], many of them are not conformed to the standard configurations, thus substantially lacking high-throughput analysis capability. The only prevailing multi-well format available to stretch experiments, to our knowledge, is a 6-well-based plate [22–25], the bottom elastic substrates of which are displaced upward/downward with a vacuum or a rigid 6-post array, thus imposing only radial but not uniaxial strains to cells. Such radial stretching always involves biaxial strains, while the capability of imposing uniaxial strains is necessary to investigate how cells sense the direction of applied stretch.

Here, we describe a new 96-well cell culture plate made of elastic silicone uniaxially stretchable with a motorized device. We demonstrate the capability of the deformable multi-well plate to allow for long-term cell culture, in which cells exhibit adaptive reorientation of their morphological polarization in response to cyclic exogenous stretch, i.e. typical cellular response. We expect this novel system to be utilized for future comprehensive analyses of stretch-related signaling pathways with the aid of other existing multi-well-based technologies such as siRNA/shRNA/drug libraries to facilitate high-throughput screening.

## Materials and methods

### Cell culture

Rat embryonic aortic smooth muscle cell lines, A7r5 cells (CRL-1444; ATCC), were cultured in DMEM (Thermo Fisher Scientific) supplemented with 10% FBS (SAFC Biosciences) and 1% penicillin/streptomycin (Wako). Cells were plated on cell culture flasks for passage culture or on lab-built 96-well plates for long-term cell culture tests, both of which were placed in a humidified 5% CO_2_ incubator at 37°C.

### Elastic cell culture plate

**Fig 1:**
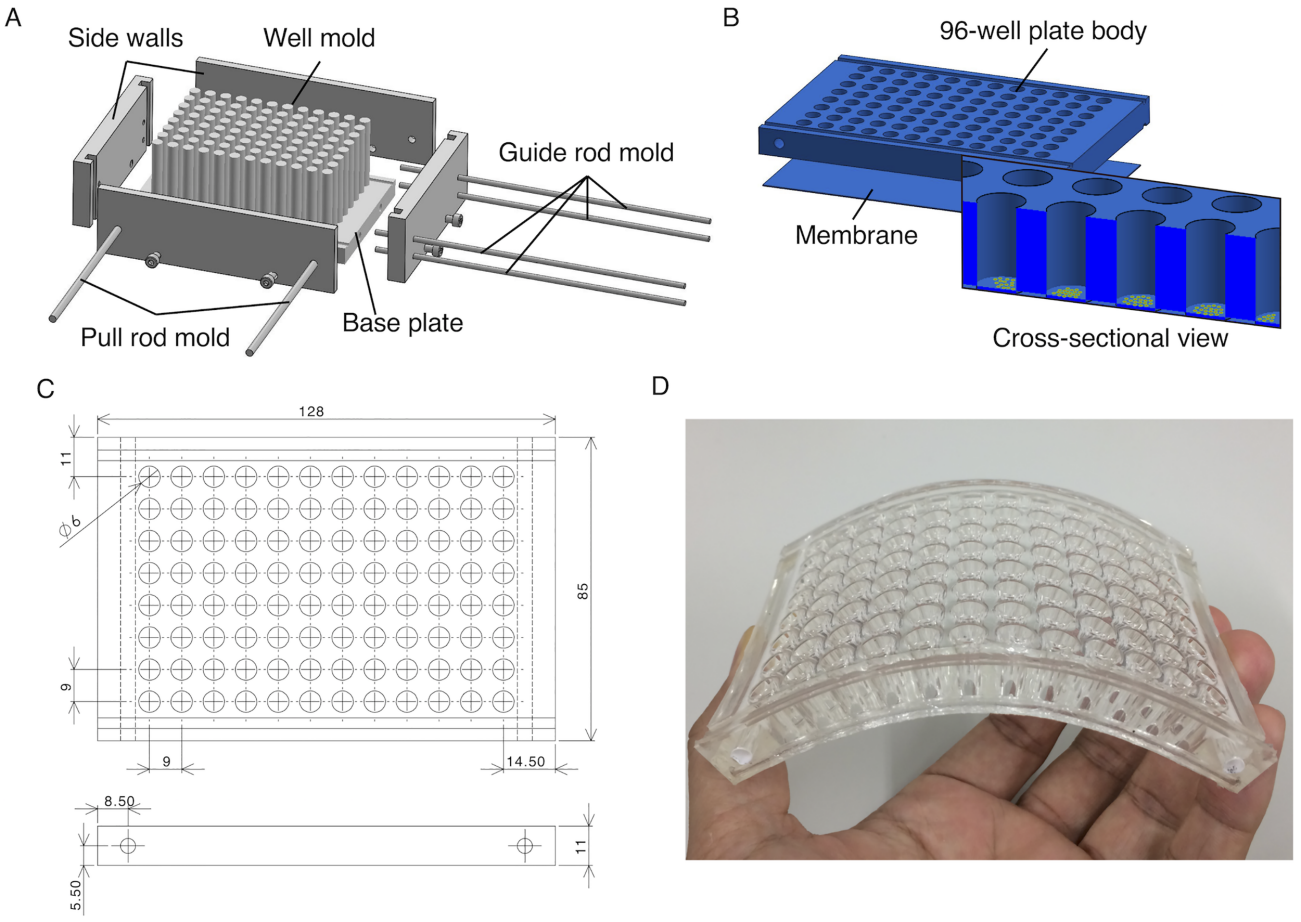
Deformable 96-well cell culture plate. (A) Mold for PDMS. (B) Plate body and membrane, both of which are made of PDMS, are combined to create the whole elastic 96-well plate. (C) Dimensions of the plate in mm. (D) Photograph of the plate.

An elastic 96-well cell culture plate was fabricated by replica molding from silicone elastomers (polydimethylsiloxane or PDMS, Sylgard 184; Toray Dow Corning) that are deformable, transparent, gas-permeable, and biologically compatible. The mold was made of 96 independent stainless-steel pillars with a cross-sectional diameter of 6 mm, which were fixed by screw-nuts at a stainless-steel bottom plate and were surrounded by acrylic sidewalls (Fig 1A and 1B). The distances between the centers of the pillars (9 mm) and between the insides of the sidewalls (128 mm in width and 85 mm in depth) were determined to fabricate a standard-compliant 96-well plate (Fig 1C and 1D). Rod-shaped holes were created along the shorter edges of the molded PDMS plate using other rod molds to afterwards insert stainless-steel pull rods. In addition, half-cylindrical grooves were created on the top and bottom surfaces along the longer edges of the molded PDMS plate using other rod molds to afterwards mount stainless-steel guide rods. These guides were aimed at minimizing the shrink of the elastic plate to the transverse direction, a behavior called Poisson’s effect, which occurs upon longitudinal stretch, and consequently at providing spatially uniform uniaxial strains at individual wells. For the preparation of PDMS, the base polymer and the cross-linker of Sylgard 184 were mixed at a weight/weight ratio of 20. After degassing in a desiccator, the uncured PDMS was casted into the mold to have a plate height of 11 mm and then oven cured at 60°C for 20 h. Separately, PDMS membranes were created using a spin coater (K-359S1; Kyowa Riken) with a lab-built acrylic circular disk to have a thickness of 200 μm. Before curing the PDMS membrane, the perforated plate was carefully placed onto the bare membrane, and the whole structure was incubated at 60°C for 20 h to enhance the bonding between them. The PDMS plate supporting the membrane was then peeled off from the acrylic circular disk. To functionalize the PDMS membrane where cells are to be plated, each well was treated with 0.5% (3-aminopropyl) trimethoxysilane (APTMS; Sigma Aldrich) in distilled water for 30 min.

### Stretch device

**Fig 2:**
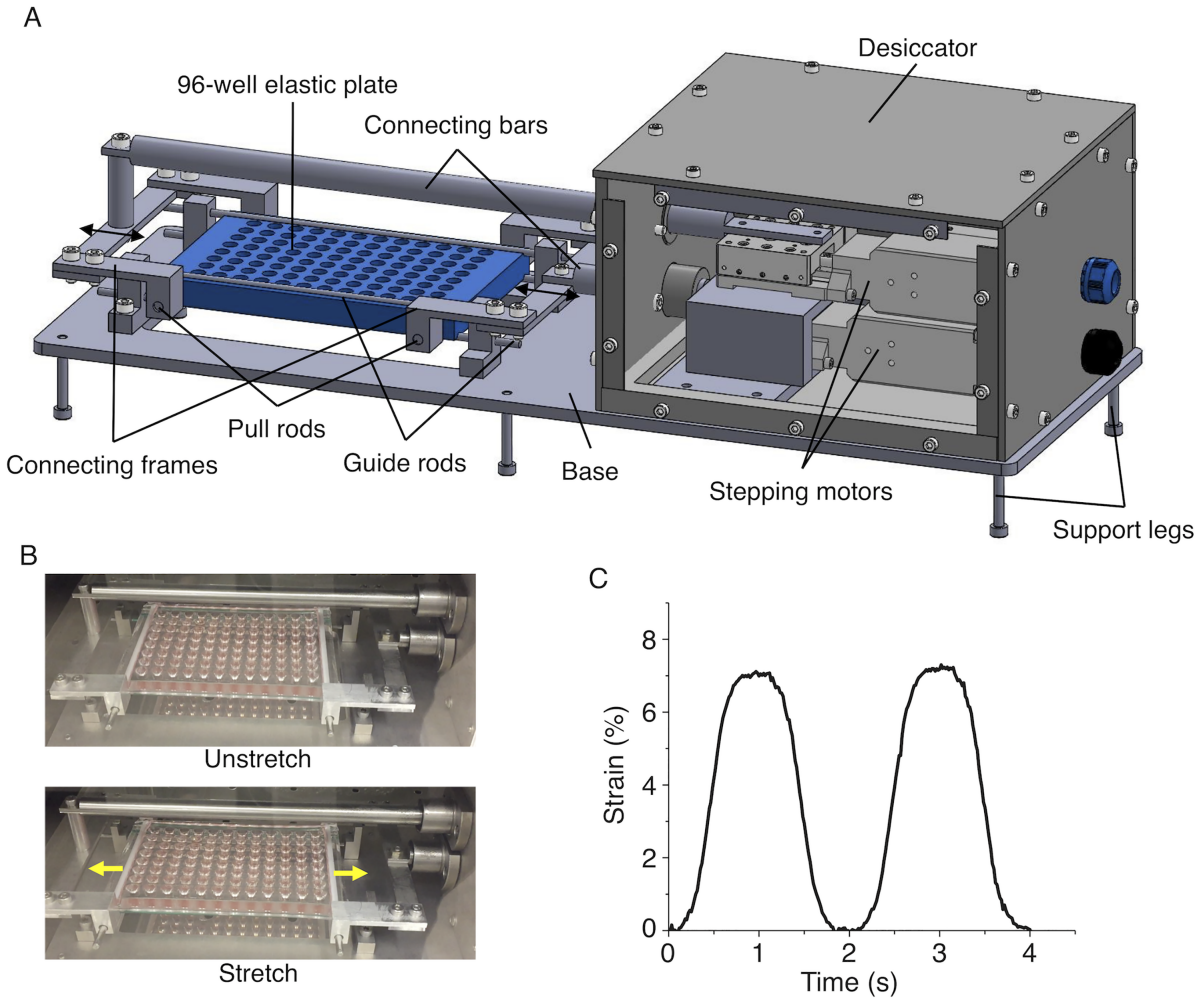
Stretch device. (A) Schematic of the device supporting the elastic plate. (B) Photographs of the elastic plate without (Unstretch) and with (Stretch) 7% stretch. (C) Temporal change in the macroscopic strain of the entire plate subjected to 7% cyclic stretch at 0.5 Hz.

Two stepping motors (with each movable in a range of 10 mm, TAMM40–10C; Sigma Koki) and a driver (SHOT-102; Sigma Koki) controlled with a lab-made program written in LabVIEW (National Instruments) were used to stretch the elastic plate up to 20 mm (Fig 2A). The axes of the motors were connected to the plate via connecting frames and pull rods that move in antiparallel directions. Four guide rods were mounted on the top and bottom sides of the plate edges along the long axis to minimize the lateral shrink accompanied by uniaxial stretch. Because the distance between the inner sides of the pull rod holes is 108 mm, our setup was designed to apply a maximum strain up to 18% (≈ 20/108 x 100). To protect the motors from moisture in a humidified cell culture incubator, they were mounted within a silicone sealant-sealed duralumin box thus acting as a desiccator. We programmed the motors to move in a sinusoidal manner.

### Finite element analysis

**Fig 3:**
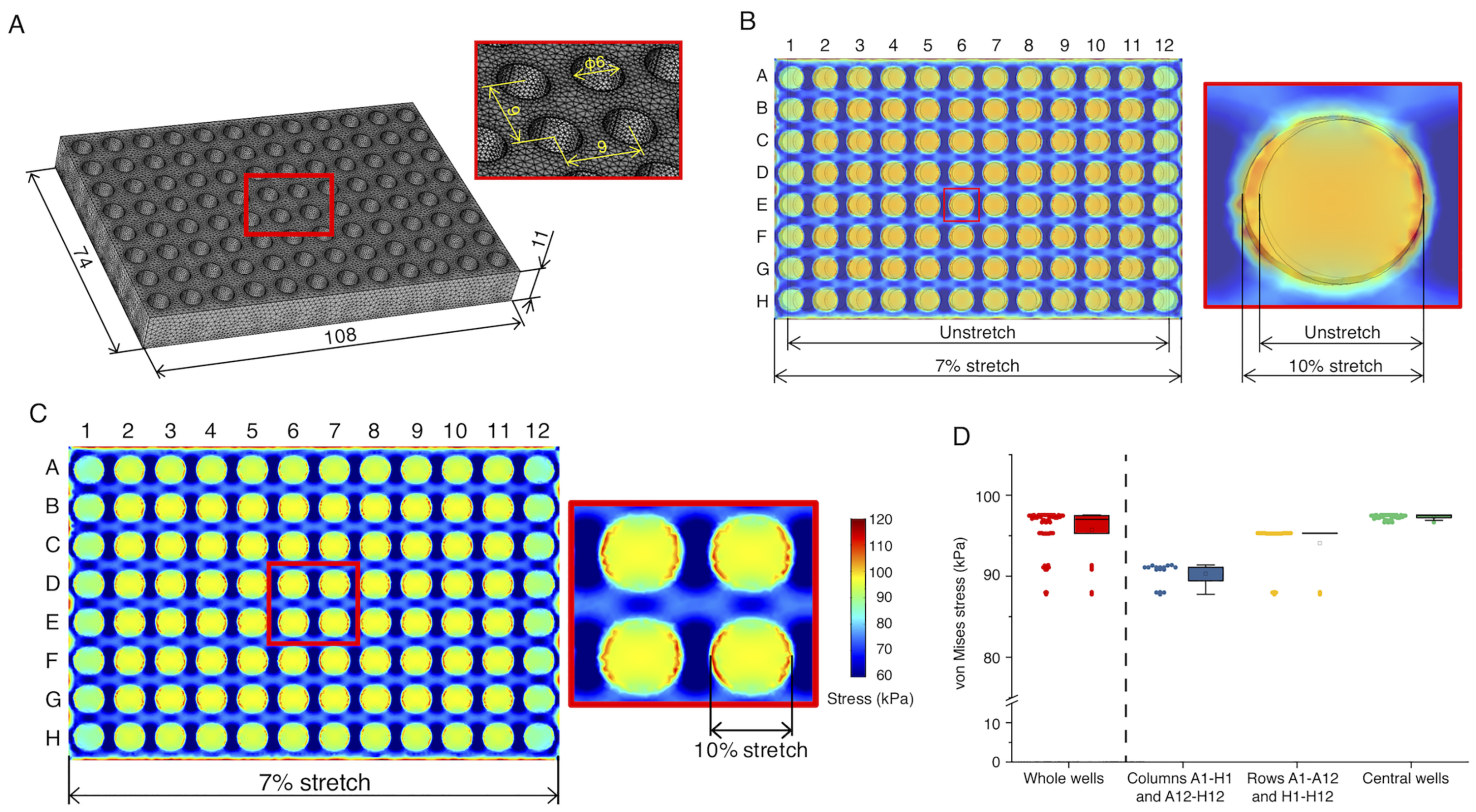
FEM analysis. (A) Dimensions of the computational plate model. (B) Merging of the images with (“7% stretch” in the left picture or “10% stretch” in the right picture) and without (Unstretch) uniaxial stretch in the longitudinal direction suggests that the macroscopic strain of 7% (exerted at the shorter plate edges) results in more microscopic strain of 10% at the individual well level. Note that the colors have no meaning here. (C) Distribution of von Mises stress generated at the bottom of the plate subjected to the macroscopic strain of 7% (i.e., 10% stretch for the individual wells). (D) Plots of von Mises stress at different regions of the plate. Boxes represent the 25th and 75th percentiles and the median. Open squares indicate means. Whiskers extend from the ends of the box to the most remote points excluding the outliers defined as 1.5 x the interquartile range.

Because the elastic plate is heterogeneous in geometry with 96 concave wells and may thus exhibit complicated stress distributions even upon simple uniaxial stretch, we assessed based on a finite element method (FEM) the relationship between the local (well; i.e., the place of cell plating) and global (plate edge; i.e., the place of pull rods) deformations. Three-dimensional FEM was performed using Comsol Multiphysics (ver. 5.2a), in which a computational model of the plate with dimensions identical to those of the actual one was built including the membrane thickness of 200 μm (Fig 3A). Young’s modulus and Poisson’s ratio of PDMS were set to be 1 MPa and 0.45, respectively, that are of typical values [26]. Mechanical stress generated within the plate was visualized with a scalar form of von Mises stress, so-called “equivalent” stress, defined as

$$\sigma_{M}=\sqrt{\{{\left(\sigma_{1}-\sigma_{2}\right)}^{2}+{\left(\sigma_{2}-\sigma_{3}\right)}^{2}+{\left(\sigma_{3}-\sigma_{1}\right)}^{2}\}/2}$$ (1)

where *σ*_1_, *σ*_2_, and *σ*_3_ are the principal stresses. Mesh sensitivity test was separately performed to ensure that the results are independent of the size of the computational meshes; in the present case, sufficiently fine mesh sizes (554,100 tetrahedron elements) were employed.

### Experimental evaluation of the plate

To evaluate the actual strain, the whole PDMS plate subjected to 0.5-Hz cyclic stretch was observed with a CMOS camera (Fig 2B). From the sequential images, the strains in the axial direction were analyzed using a program written in LabVIEW and Vision software (National Instruments).

**Fig 4:**
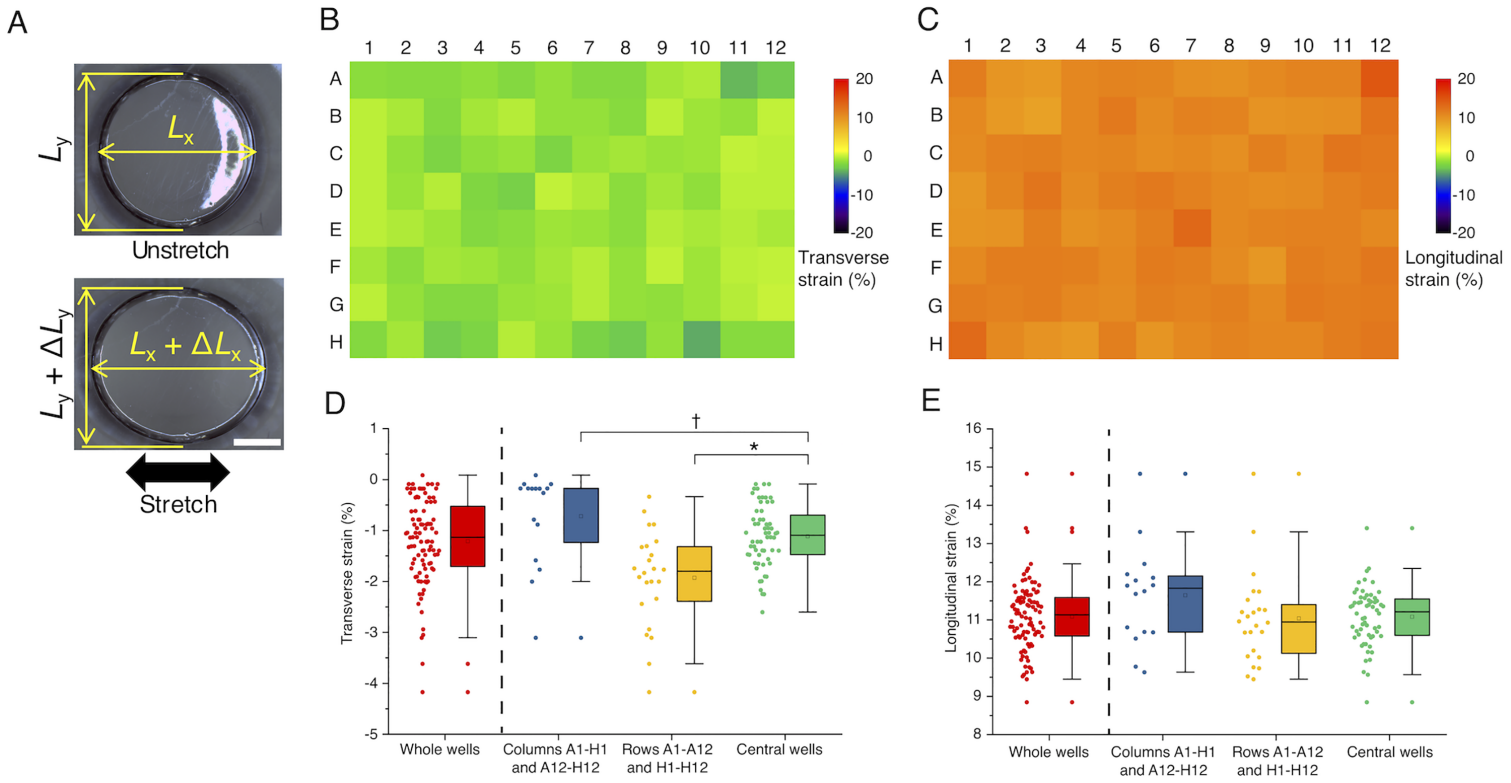
Measurement of strains based on changes in the diameter of wells. (A) Photographs of an identical well before (Unstretch) and after (Stretch) 10% uniaxial stretch. Black arrow represents the direction of stretch. Scale bar, 2 mm. (B) Map of transverse strains at each well position. (C) Map of longitudinal strains at each well position. (D) Transverse strains at different regions of the plate. (E) Longitudinal strains at different regions of the plate. Representative data from *N* = 2 independent experiments and analyses. Boxes represent the 25th and 75th percentiles and the median. Open squares indicate means. Whiskers extend from the ends of the box to the most remote points excluding outliers. †, *P* < 0.01 by Welch’s test assuming unequal variance with rejection of outliers; *, *P* < 0.001 by Student’s *t*-test.

In a separate experiment, the deformations of individual wells upon the 7% stretch exerted on the shorter plate edges were monitored with stereomicroscopy (S8APO; Leica) (Fig 4A). Changes in the maximum diameters were measured for each well along the major (*x*) and minor (*y*) axes between the unstretch (*L*_x_ and *L*_y_, respectively) and stretch (*L*_x_ + Δ*L*_x_ and *L*_y_ + Δ*L*_y_, respectively) conditions. Longitudinal strain in the major axis and transverse strain in the minor axis were determined for each well as 100Δ*L*_x_/*L*_x_ (%) and 100Δ*L*_y_/*L*_y_ (%), respectively.

**Fig 5:**
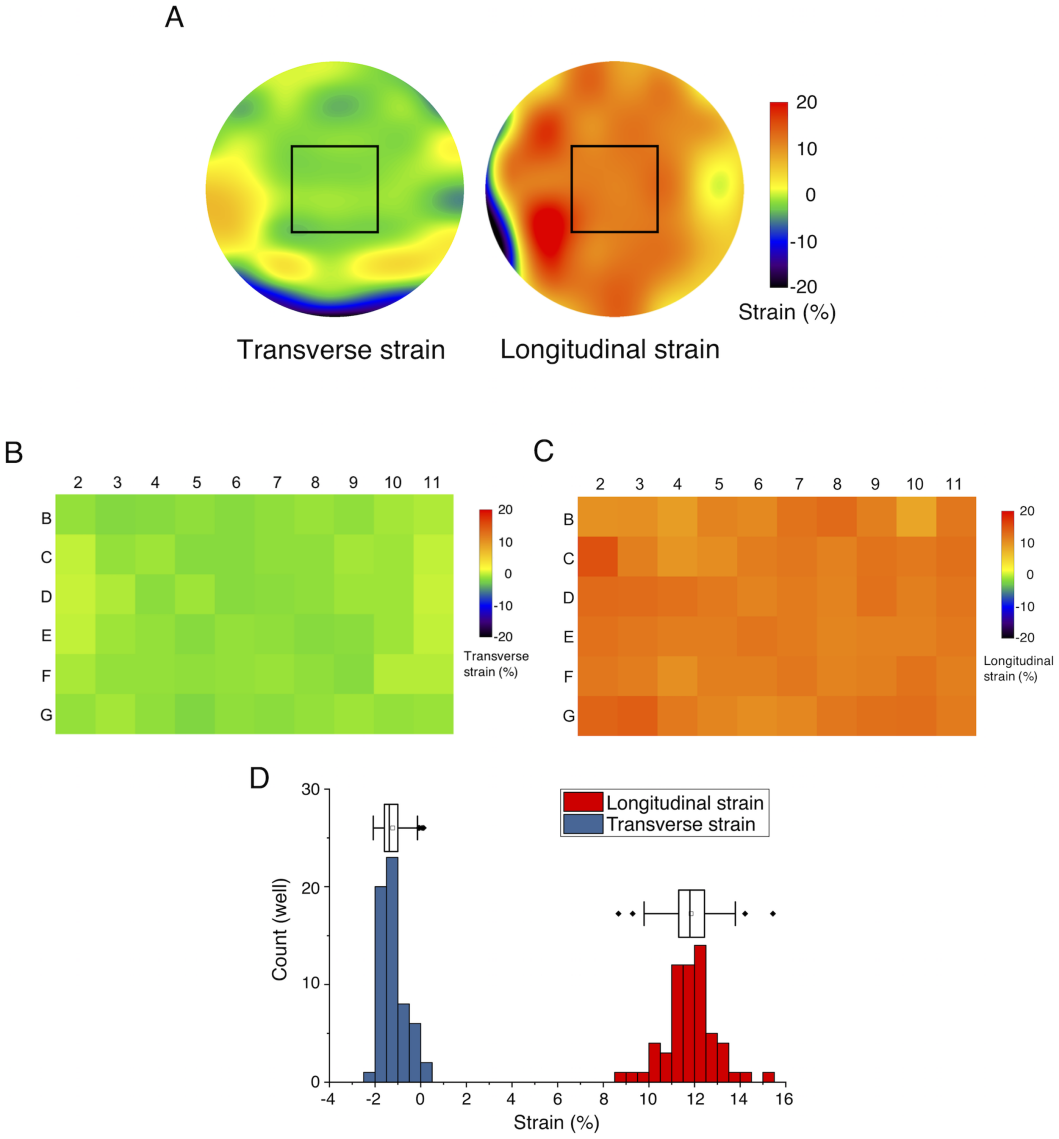
Elastic image registration to evaluate local strain distributions within individual wells. (A) Representative data of local strain distributions within a well under macroscopic 7% stretch exerted on the shorter plate edges. Rectangular regions were analyzed for the subsequent B—D and imaged for cell experiments. (B) Map of transverse strains. (C) Map of longitudinal strains. (D) Frequency distribution of the magnitude of the strains. Boxes represent the 25th and 75th percentiles and the median. Open squares indicate means. Whiskers extend from the ends of the box to the most remote points excluding outliers.

Furthermore, elastic image registration was performed using ImageJ/Fiji plugin bUnwarpJ [27] to evaluate local strain distributions within individual wells. For this analysis, ~20 different points were selected from fiducial markers on the PDMS membranes for each well to determine landmark constraints. Under the constraints, an energy function that includes the dissimilarity between before and after the macroscopic 7% stretch exerted on the shorter plate edges was minimized to determine the local elastic deformations represented by B-splines. Spatial distributions of the resulting longitudinal and transverse strains were then obtained (Fig 5A).

### Long-term cell culture test

**Fig 6:**
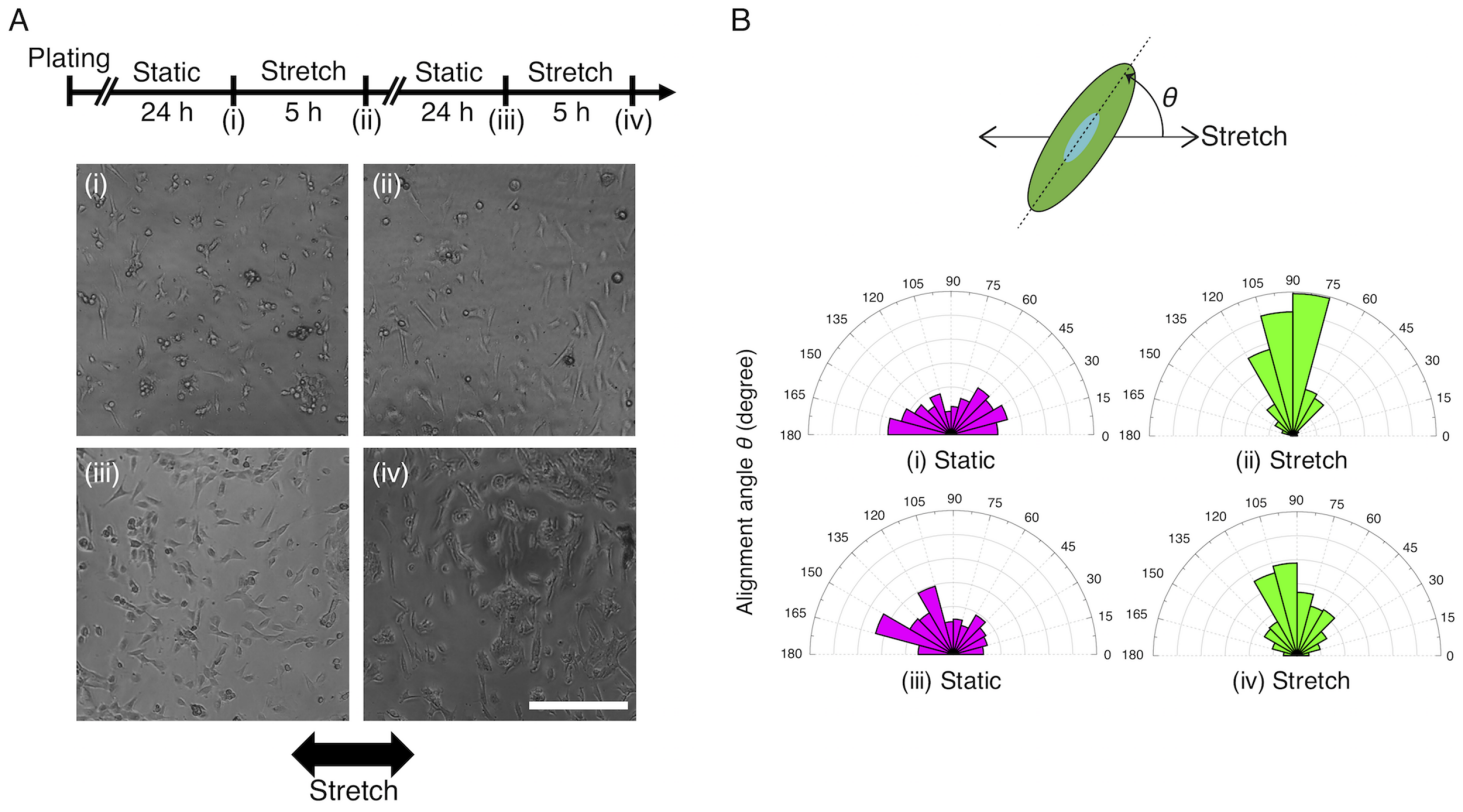
Long-term cell culture test demonstrates repeatable adaptive response of cells to cyclic uniaxial stretch. (A) Experimental time course and representative cell images. Black arrow represents the direction of the stretch. Scale bar, 300 μm. (B) Wind rose histograms of the cell alignment angle at the time points (i)–(iv) shown in A. The number of cells; (i) *n* = 226, (ii) *n* = 232, (iii) *n* = 211, and (iv) *n* = 311.

To demonstrate the capability of the new elastic plate to allow for long-term cell culture, A7r5 cells were cultured on the wells initially without stretch (Static) and imaged on a phase-contrast microscope (CKX41; Olympus) at 24 h after plating (i, Fig 6A). Individual cells were subsequently exposed to the macroscopic 7% stretch at the level of the entire plate at 0.5 Hz for 5 h and then imaged by microscopy (ii). Cells were again subjected to static culture for 24 h and then imaged (iii). Finally, individual cells were again exposed to the cyclic stretch at 0.5 Hz for 5 h and then imaged (iv). During this test, cells were placed in the CO_2_ incubator except for the imaging process to perform microscopy. To characterize the cellular response, the contour of cells was manually traced and approximated to an ellipse using ImageJ/Fiji software (Fig 6B). The angle between the stretch direction and the long axis of the fitted ellipse, *θ*, was quantified to evaluate the alignment of cells.

## Results

### FEM predicts uniform deformation of individual wells

We computationally simulated with FEM the deformation of the designed 96-well plate. Because 10% stretch is often used in conventional studies on cellular response to stretch, we aimed at achieving 10% to be applied to cells. From the analysis, we found that macroscopic 7% stretch exerted on the shorter plate edges results in microscopic 10% stretch at the level of individual wells because the deep concave (i.e., holed) portions are more prone to be stretched compared to the convex portions (Fig 3B). For this analysis, we assumed that the guide rods perfectly work to suppress—along the longer edges—the lateral shrink accompanied inevitably by the uniaxial stretch, i.e. Poisson’s effect. Because resulting slight lateral pulls were induced inside of the plate, stress was accordingly increased there other than the outermost wells of the plate. To more specifically evaluate the spatial difference between the central and peripheral portions, von Mises stress was analyzed over the entire plate (Fig 3C). The location of individual wells in a 96-well plate format is typically specified by a combination of numbers (1–12 for column) and letters (A—H for row). Representative von Mises stress values were determined at each of the centers of the individual wells (where the stress is almost spatially uniform) and categorized into three depending on the location of the wells: i.e., i) “Columns A1–H1 and A12–H12” (that have 16 wells in total), ii) “Rows A1–A12 and H1–H12” (that have 24 wells in total), and finally iii) “Central wells” that represent the wells in the rectangular region surrounded by B2–B11–G11–G2 (that have 60 wells in total). The stress was highly uniform within “Central wells” with a mean of 97.3 kPa and a standard deviation (SD) of 0.3 kPa thus with a small SD—mean ratio (Fig 3D). Meanwhile, the stresses at the outermost areas (“Columns A1–H1 and A12–H12”, 90.3 ± 1.4 kPa (mean ± SD); “Rows A1–A12 and H1–H12”, 94.1 ± 2.8 kPa) were relatively low and varied in magnitude compared to “Central wells” area.

### The actual strains partly vary but reach a target value

To evaluate the actual strain of the plate in response to motions of the motors connected via several mechanical links, the entire plate was imaged during cyclic stretching (Fig 2B). Quantification of the captured sequential images indicated that indeed the entire plate exhibited a desired sinusoidal 7% stretch pattern as consistent with the programed motions of the motors (Fig 2C).

Next, we evaluated strains at the individual well level by microscopy. First, we mapped the longitudinal and transverse strains of wells in the major and minor axes, respectively, by measuring the difference in the outermost edges (Fig 4B and 4C). These quantitative data indicated that the longitudinal strains reached, upon the macroscopic 7% stretch exerted on the shorter plate edges, 11.6% ± 1.3% (mean ± SD) for “Columns A1–H1 and A12–H12”, 11.0% ± 1.2% for “Rows A1–A12 and H1–H12”, and 11.1% ± 0.8% for “Central wells” (Fig 4E). Thus, we found that the longitudinal strains obtained in the experiments can be variable and exceed on average the theoretical value 10%, but the magnitude was almost comparable to the prediction by FEM. The transverse strains in the same condition were -0.7% ± 0.9% for “Columns A1–H1 and A12–H12”, -1.9% ± 0.9% for “Rows A1–A12 and H1–H12”, and -1.1% ± 0.6% for “Central wells” (Fig 4D). Here, a statistically significant difference was present between central and peripheral wells (Fig 4D) so that in the following local strain analyses we focused only on the central wells.

Spatial distributions of the strains in individual wells were measured by elastic image registration (Fig 5). Central regions of the wells where cells are to be imaged by microscopy (Fig 5A, rectangles) were almost uniform in strain magnitude. Mapping the longitudinal and transverse strains averaged for each of the rectangles (Fig 5B and 5C) showed that the tendency was comparable to the results quantified from the difference in the well diameter (Fig 4D and 4E). Specifically, the local longitudinal and transverse strains were 11.8% ± 1.1% and -1.2% ± 0.5%, respectively (Fig 5D).

### The device is applicable to long-term cell culture

We observed that cells cultured in the elastic plate exhibited typical response to cyclic stretch to orient away from the direction of stretch (Fig 6A). The cellular response to stretch is known to be repeatable because cells constantly sense changes in their mechanical environment and thus return to their original morphology with no preference in orientation upon static culture. This reversible cellular response was observed for over two days, demonstrating the capability of our device allowing for sufficiently long-term cell cultures (Fig 6B). The present test also confirmed that the bottom of the PDMS membrane was attached to the plate body without leakage of culture media even during the whole process of the stretching.

We separately confirmed that cells on the PDMS membrane can be detected by epifluorescence microscopy with fluorescent phalloidin staining for F-actin (S1 Fig). In conventional fluorescence plate readers, epifluorescence images are taken so that the side walls of the plate do not affect the microscopy if an inverted objective lens is used. The thickness of the bottom PDMS membrane measured was less than 200 μm, and a cover glass attached under the membrane was 50 μm in thickness. Thus, epifluorescence images can be acquired even via a high-magnification objective of 60x with a short working distance (150 μm for a 170-μm-thickness glass). It will be easier to image cells with lower magnification objectives with longer working distances such as 40x and 20x.

## Discussion

Here, we developed the deformable 96-well cell culture plate with the future perspective of coupling with other 96-well-based technologies such as siRNA/shRNA/drug libraries, automated fluorescence plate readers, and micro-channel pipettes. Given its universal design, we expect our system to be utilized, efficiently together with such existing technologies, as a platform allowing for comprehensive analyses of cellular mechanobiological processes.

During the preparation of our paper, we noticed a 96-well cell culture plate with a silicone membrane attached to the bottom of the rigid plate body has been reported to evaluate the effect of neuronal stretch injury [28]. In this setup, the membrane is stretched in all directions by upward displacement of an underneath rigid post array, thus incompatible with analyzing the effect of uniaxial stretch on the cellular repolarization into the specific direction. In addition, large variations in stress may be generated with this method over the area of individual wells, thus complicating the interpretation of data if cell lysis is performed. For other mechanical forces other than stretch, we noticed multi-well plate-based devices have been recently reported regarding shear stress [29–31]. Such high-throughput-oriented technologies developed in conformity with the standard plate geometries can advance a new approach in the field of mechanobiology with an “omics” viewpoint or “mechanomics”.

Cellular response to stretch has been extensively studied, but conventional experimental systems are often constituted from single or only 6 wells partly because the majority of the previous studies focused on imaging of the dynamics of specific individual cells/molecules [13,18,32–37] or examining changes in mRNA expression [22,38] rather than performing an assay for molecular screening, the last of which generally requires huge numbers of screening trials. For example, a screening study was recently reported to reveal the Rho-GEFs responsible for the cyclic stretch-induced repolarization from 63 candidate molecules [6]. Here, they employed a stretch chamber with a single well of a 20 x 20 mm^2^ cell culture area and a Rho-GEFs-targeted shRNA library, which we guess might took long time to complete and was costly to prepare large amounts of reagents. In this regard, our strategy of combining the stretch chamber and library directly at the small individual well levels can highly improve the screening throughput and cost. In addition to such candidate gene screenings, our system is useful as well in compound screening with various reagent libraries to suppress or rescue cellular responses altered by gene mutations.

Previous multi-well plates with an elastic membrane attached to the bottom of the rigid plate body (i.e., a 96 well plate [28] or a 6 well plate [22–25,38]) allow for loading of radial (or biaxial) stretch with upward displacement of an underneath rigid post array or vacuuming. On the other hand, realizing uniaxial stretch under multi-well formats is technically difficult, while uniaxial stretch is widely present in tissues throughout the body including brain tissues [39] and thus important to study the cellular response. We mounted in our setup four guide rods to minimize the lateral shrink that impairs the application of uniaxial stretch, although they could not partly suppress the inevitable Poisson’s effect. The guide rods worked relatively well near the shorter edges of the plate, and consequently the compressive strains were relatively low there (Fig 4D). In addition, some variations, larger in magnitude compared to the theoretical values from FEM (Fig 3), appeared in all directions probably because of manufacturing defects (Fig 4). We initially designed and fabricated a similar elastic plate with its well’s shape being square in top view because the strain distribution within individual wells is ideally further uniform compared to that within circular-shaped wells. However, we found in preliminary experiments that manufacturing defects were formed more obviously in the case of the square-well plate; thus, we decided to use more reliable circular wells in the present study. Another reason for the difference between the theoretical and experimental results includes the too restricted boundary conditions of the FEM model. The presence of the variations in applied strain level must be considered as a limiting factor; nevertheless, our device will be useful to enable high-throughput screening of molecules that change the activity depending on the presence or absence of uniaxial stretch.

To enhance cell adhesion, we employed silane coupling using APTMS. In PDMS-based bioreactors, the initially hydrophobic PDMS is often functionalized with oxygen plasma to enhance cell adhesion [40]. Yet, we found in preliminary studies that such plasma treatment is not suitable for the present case because highly brittle glass-like layers are produced, and cracks are inevitably created on the fragile layer even with subtle stretching to consequently degrade the image quality of automated image analyses necessary for high-throughput screening [41]. With the APTMS-treated substrate, we did not detect in phase-contrast microscopy cracks after cyclic stretching, thus suggesting that APTMS-treated wells function as a cell culture substrate that is mechanically endurable even with the challenge of cyclic stretch.

## Supporting information

S1 Fig Typical fluorescence image of a cell stained with fluorescent phalloidin. (A) The side view of the imaging system. (B) A7r5 cell line labeled with fluorescent phalloidin plated on a well of the plate. Scale, 30 μm.
(TIFF)
