# Supplementary figures and images for: Deformable 96-well cell culture plate compatible with high-throughput screening platforms
Source: PLoS One. 2018 Sep 6;13(9):e0203448. doi: 10.1371/journal.pone.0203448 (PMC6126838; doi:10.1371/journal.pone.0203448)

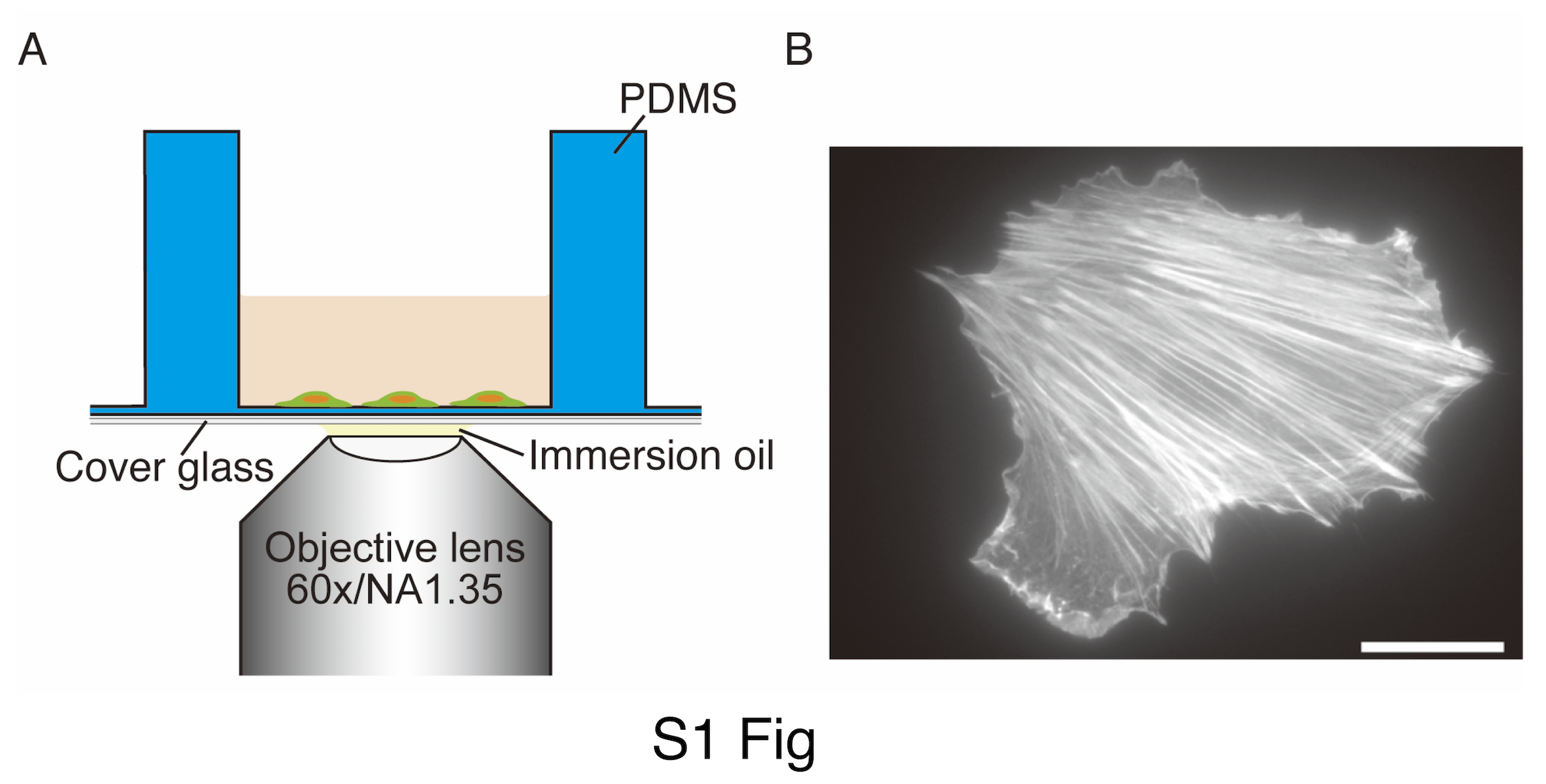

Supplement: S1 Fig — (A) The side view of the imaging system. (B) A7r5 cell line labeled with fluorescent phalloidin plated on a well of the plate. Scale, 30 μm. (TIFF) [file pone.0203448.s001.tiff]
